# Genome-Scale Analysis of *Mycoplasma agalactiae* Loci Involved in Interaction with Host Cells

**DOI:** 10.1371/journal.pone.0025291

**Published:** 2011-09-23

**Authors:** Agnès Skapski, Marie-Claude Hygonenq, Eveline Sagné, Sébastien Guiral, Christine Citti, Eric Baranowski

**Affiliations:** 1 INRA, UMR1225, IHAP, Toulouse, France; 2 Université de Toulouse, INP, ENVT, UMR1225, IHAP, Toulouse, France; Institut de Pharmacologie et de Biologie Structurale, France

## Abstract

*Mycoplasma agalactiae* is an important pathogen of small ruminants, in which it causes contagious agalactia. It belongs to a large group of “minimal bacteria” with a small genome and reduced metabolic capacities that are dependent on their host for nutrients. Mycoplasma survival thus relies on intimate contact with host cells, but little is known about the factors involved in these interactions or in the more general infectious process. To address this issue, an assay based on goat epithelial and fibroblastic cells was used to screen a *M. agalactiae* knockout mutant library. Mutants with reduced growth capacities in cell culture were selected and 62 genomic loci were identified as contributing to this phenotype. As expected for minimal bacteria, “transport and metabolism” was the functional category most commonly implicated in this phenotype, but 50% of the selected mutants were disrupted in coding sequences (CDSs) with unknown functions, with surface lipoproteins being most commonly represented in this category. Since mycoplasmas lack a cell wall, lipoproteins are likely to be important in interactions with the host. A few intergenic regions were also identified that may act as regulatory sequences under co-culture conditions. Interestingly, some mutants mapped to gene clusters that are highly conserved across mycoplasma species but located in different positions. One of these clusters was found in a transcriptionally active region of the *M. agalactiae* chromosome, downstream of a cryptic promoter. A possible scenario for the evolution of these loci is discussed. Finally, several CDSs identified here are conserved in other important pathogenic mycoplasmas, and some were involved in horizontal gene transfer with phylogenetically distant species. These results provide a basis for further deciphering functions mediating mycoplasma-host interactions.

## Introduction

The term “mycoplasma” is used trivially to describe bacteria belonging to the class *Mollicutes*, which includes the genus *Mycoplama*, as well as several other related genera [1]. These micro-organisms have evolved from a low G+C content Gram positive ancestor by “regressive evolution”, resulting in massive genome reduction [2], [3]. As a result, contemporary mycoplasmas lack a cell-wall and are commonly described as the smallest self-replicating organisms, because of the small size of their genome (580 to 1,400 kbp) and the paucity of their metabolic pathways. Mycoplasmas and ureaplasmas live in close contact with animal tissues, probably because of their limited metabolic capacity, a feature that is likely to have increased their dependence on hosts for a number of nutrients [2]. Mycoplasmas occur widely in nature and, despite their apparent simplicity, several species are successful pathogens of animals, in which they establish persistent infections and cause chronic disease [4].

*Mycoplasma agalactiae* is an important pathogen of small ruminants that causes contagious agalactia (CA), resulting in significant losses in the sheep and goat milk industries [5]. It is classified by the World Organization for Animal Health (OIE) as a notifiable disease and the clinical signs include mastitis, arthritis and kerato-conjunctivitis [5]. Contagious agalactia is also caused by several members of the mycoides cluster, including *M. mycoides* subspecies *mycoides* Large Colony type and *M. capricolum* subspecies *capricolum* [5]. Interestingly, while these mycoplasmas are phylogenetically distant from *M. agalactiae*, detailed *in silico* genomic analyses have revealed that extensive horizontal gene transfer has occurred between *M. agalactiae* and members of the mycoides cluster, and as a result these mycoplasmas may share a number of common cell surface functional domains [3], [6]. Phylogenetically, *M. agalactiae* is closely related to *M. bovis* [7], a pathogen of large ruminants that causes clinical signs similar to those of contagious agalactia [8]. For all these ruminant mycoplasmoses, the factors involved in colonization, dissemination and pathogenicity are poorly understood. As a number of genetic tools and genomic data are available for *M. agalactiae* [6], [9], [10], this species is a useful model for studying the molecular players involved in infectious processes and thus furthering comprehension of pathogenic mechanisms in other mycoplasmas.

A common approach used to identify virulence genes in pathogenic bacteria is based on random transposon mutagenesis [11]–[13]. In mycoplasmas, this approach has mainly been applied to study the minimal set of essential genes [14]–[16], but has also been successfully employed in a few cases to identify genes potentially involved in pathogenicity [17], gliding motility and adherence [18], [19]. Such an approach is needed to further understanding of *M. agalactiae*, as *in silico* analyses of currently available ruminant mycoplasma genomes has failed to reveal unambiguously loci that might contribute to infection. Indeed, predicted *M. agalactiae* gene products have little to no similarity to virulence factors known in other bacteria, and 40% of the coding sequences (CDSs) have been annotated as hypothetical proteins with unknown functions [6]. For *M. agalactiae* and other ruminant species, one limitation of global transposon mutagenesis to identify virulence genes is the absence of a small laboratory animal model of infection, as *in vivo* screening in the natural ruminant hosts is constrained by both technical and ethical problems. To overcome these issues, we developed a method for high-throughput screening of *M. agalactiae* knockout mutants by co-cultivating *M. agalactiae* mutants with HeLa cells [20]. This assay allowed the selection of a number of genomic regions potentially required for growth in HeLa cell cultures, but dispensable in axenic conditions. Human epithelial surfaces are not a natural environment for *M. agalactiae*, so we extended our functional genomic study by using two caprine cell lines that are more relevant to the natural host context: goat mammary epithelial cells, TIGMEC, which are likely to be good targets based on the predilection of *M. agalactiae* for the mammary gland, and goat embryo fibroblasts, TIGEF.

Over 2000 *M. agalactiae* mutants were co-cultured with caprine cells and those showing a significant reduction in their capacity to grow were examined in detail, revealing 62 loci potentially required for propagation in the host environment. The relevance of these loci and the potential role of the genes at these loci in *M. agalactiae*-host interactions were analyzed.

## Materials and Methods

### Bacteria, cell lines and culture conditions

*M. agalactiae* reference strain PG2 (Refseq NC_009497) [6] was grown in Aluotto or SP4 medium as described previously [20]. Titers were determined by serial dilution in Dulbecco's phosphate-buffered saline (Invitrogen) containing 1% heat-inactivated horse serum (Invitrogen). *E. coli* DH10B (Invitrogen) was used for DNA cloning and plasmid propagation. The human and caprine cell lines used in this study included HeLa cells (ATCC CCL2), SV40 large T-antigen immortalized goat embryo fibroblasts (TIGEF) and similarly immortalized goat mammary epithelial cells (TIGMEC). TIGMEC were derived from milk epithelial cells [21]. Immortalized cells exhibited morphological and phenotypic features of parental milk epithelial cells and expressed cytokeratin, a specific marker of epithelial cells. Immortalized goat embryo fibroblasts were generated from carpal synovial membrane explants and displayed morphological features of fibroblastic cells [22]. Cells were grown in Dulbecco's modified Eagle's medium (DMEM)-based medium, as described previously [20], composed of DMEM (high glucose, sodium pyruvate, and GlutaMAX-I; Invitrogen) supplemented with non-essential amino acids (NEAA; Invitrogen) and 10% heat-inactivated fetal calf serum (FCS; Eurobio).

### *M. agalactiae* knockout mutant library

The library of knockout mutants was produced in *M. agalactiae* reference strain PG2 [20]. Transposon mutagenesis was carried out using plasmid pMT85, which does not replicate in mycoplasmas, but contains a modified version of transposon Tn*4001* (mini-Tn) conferring gentamicin resistance [23]. Mutants were collected from individual transformations to produce a representative library of 2,175 individual mutants. The pMT85-based library was propagated in SP4 medium supplemented with 500 µg cephalexin/ml (Virbac) and 50 µg gentamicin/ml (Invitrogen). Transposon insertion sites in the *M. agalactiae* chromosome were mapped by direct sequencing of the junction between the *M. agalactiae* genomic DNA and the 3′ end of the transposon using BigDye Terminator chemistry and oligonucleotide primer SG8 (Table 1). Direct sequencing was performed at the sequencing facility at the Bio-Medical Research Federative Institute of Toulouse (Toulouse, France). The 3′ end of the mini-Tn was defined using the orientation of the gentamicin resistance gene as the reference [23]. The Pip, MucB and P40 minus mutants were identified by PCR screening of the mutant library using specific oligonucleotide primers [20]. The mutants NifS1 and NifS2 have been described previously as mutants 7.82 and 7.134, respectively [20]. These mutants had a mini-Tn insertion at genomic positions 86804 (Pip), 88958 (MucB), 281483 (P40 minus), 87172 (NifS1) or 88125 (NifS2). The mini-Tn gentamicin resistance gene in the NifS1, Pip, MucB and P40 minus mutants was in the opposite orientation compared to the disrupted CDS, but in the same orientation in the NifS2 mutant.

**Table 1:** Oligonucleotides used in this study.

Name	Sequence (5′→3′)
SG5	TTTTACACAATTATACGGACTTTATC
SG8	GAGTCAGTGAGCGAGGAAGC
P40_RF_CC	ACGGGGCTAAAGAAGCTGAT
CCP40-03	TGGTTATATTTTCCATATCTTTC
Pip-P40_F	GCAATTGAGAATTTTATTAAAGGATAAATA-ATGAAAACAAATAGAAAAATATTGTTTGGT
Pip-P40_R	ACCAAACAATATTTTTCTATTTGTTTTCAT-TATTTATCCTTTAATAAAATTCTCAATTGC
Pip-86804_F	GCCAGCCATATGGTGCATATTTAG
Pip_F	TATTCGACCAAAGAGGGTGT
Pip_R	TCATCAAAATCACCACCAAG
NifS_F1	TCAGCCGACATTATTCATGG
NifS_R1	CACCGGCTTTTAATTTTTGC
NifS_F2	TGTCACAAAGTTGGAGCAAT
NifS_R2	ACGAAGGAATCGTTACAAGC
NifU_F	AGGGTTTCGCTAGGGGTTTA
NifU_R	CTGTGCGCGCTTACAAAGTA
5′-6FAM-NifS1^a^	TAGTTCTTGTGCTAACCGAATA

a Oligonucleotide labeled by a 6-carboxyfluorescein (FAM) 5′-modification.

### High-throughput screening of *M. agalactiae* knockout mutant library in cell culture

A cell-culture assay was used to screen the *M. agalactiae* knockout mutant library and identify mutants displaying a growth-deficient phenotype [20]. Briefly, cells seeded in 96-well plates were inoculated with cultures of individual mutants using a 96-pin replicator (Boekel Scientific). Growth-deficient mutants were selected based on the titers reached at the end of the co-cultivation period. After one freeze-thaw cycle (−80°C/+37°C), co-cultures were spotted onto solid medium using a 96-pin replicator. The development of mycoplasma colonies was used as a cut-off point (Table 2). Culture stocks (inoculum) of *M. agalactiae* knockout mutants were tested by direct spotting onto solid medium. The titer reached by the wild-type after co-cultivation with HeLa cells [20], TIGMEC or TIGEF cells was not influenced by variations in the initial inoculum size (data not shown). *M. agalactiae* was unable to proliferate in cell-culture medium alone [20].

**Table 2:** High-throughput detection of *M. agalactiae* mutants unable to grow on cultured cells.

Cut-off value (CFU titers)^a^	TIGMEC^b^	TIGEF	HeLa
0 (10^4^ CFU/ml)	15 (0.7%)^c^	15 (0.7%)	25 (1.1%)
10 (10^5^ CFU/ml)	26 (1.2%)	96 (4.4%)	153 (7.0%)

a The cut-off value is the number of colonies counted on solid medium following 3 days co-cultivation of *M. agalactiae* knockout mutants with cells. Titers in parentheses indicate the predicted titers in the co-culture.

b Number of *M. agalactiae* growth deficient mutants selected on goat mammary epithelial cells (TIGMEC), goat embryonic fibroblast cells (TIGEF), and HeLa cells (HeLa).

c Percentage of growth deficient mutants selected from the mutant library.

### RNA extraction, RT-PCR amplification and primer extension

Total RNA was extracted from 48 hour cultures of *M. agalactiae* using the TRIzol method (Invitrogen). RNA samples were stored at −80°C. The RNA concentration was determined spectrophotometrically by measuring absorbance at 260 nm and by agarose gel electrophoresis. RT-PCRs were carried out with the Access RT-PCR System kit (Promega), using 1 µg of total RNA treated with DNAse (RNase-free DNAse, Promega). Reactions were incubated at 45°C for 45 min, 94°C for 2 min, then through 30 cycles of 94°C for 30 sec, 56°C for 1 min and 72°C for 30 sec, with a final extension incubation of 7 min at 72°C. Amplification products were analyzed by agarose gel electrophoresis. Primer extension was carried out using 15 µg of total RNA, 2 pmol of the labeled oligonucleotide primer 5′-6FAM-NifS1 (Table 1), and 200 units of Superscript III Reverse Transcriptase (Invitrogen). RNAs were denatured by 5 min incubation at 65°C. Reverse transcription reactions were performed at 42°C for 50 min, followed by enzyme inactivation at 75°C for 15 min. The cDNAs were treated with RNAse A (Promega) for 30 min at 37°C, ethanol precipitated and resuspended in 10 µl formamide and 0.4 µl of GS-400HD ROX size standards (Applied Biosystems). Product sizes were analyzed using the ABI3730 sequencer (Applied BioSystems) at the GenoToul genomic platform of Toulouse (France).

### A reporter system for the detection of transcriptional promoter sites in *M. agalactiae*

The surface antigen P40 CDS MAG2410 was used as a reporter gene to assess transcriptional promoter activity in *M. agalactiae*. DNA sequences to be tested were cloned upstream of CDS MAG2410 using the plasmid p20-1miniO/T as a vector [20]. The 3′ end of the *pip* gene was amplified by PCR using the primers Pip-86804_F and Pip-P40_R (Table 1) to generate a 191 bp fragment overlapping CDS MAG2410. CDS MAG2410 was amplified using Pip-P40_F and CCP40-03 (Table 1) to generate a 1188 bp fragment overlapping the 3′ end of the *pip* gene. The two overlapping fragments were assembled by PCR amplification using the primers Pip-86804_F and CCP40-03 (Table 1). The resulting PCR products were cloned into the pGEM-T Easy vector (Promega), before sub-cloning into the *Not*I site of plasmid p20-1miniO/T. PCR amplifications were performed using the proofreading Phusion High Fidelity polymerase (Finnzymes). Before cloning into pGEM-T Easy, 3′-terminal deoxyadenosine residues were added to blunt-ended PCR products by following the A-tailing procedure provided by the supplier (Promega). Cloned sequences were verified by DNA sequencing. CDS MAG2410 alone or with its own promoter sequence was used as negative and positive controls, respectively. PCR amplifications were performed using primers P40_RF_CC and CCP40-03 (positive control), and Pip-P40_F and CCP40-03 (negative control)(Table 1).

A surface antigen P40-knockout mutant (P40 minus; see above) was used to test the expression of P40 from the different plasmid constructs. *M. agalactiae* cells (10^8^ to 10^9^ CFU) were transformed by electroporation using 1 to 3 µg of plasmid DNA, as described previously [20]. After 3 hours incubation in non-selective medium, cells were allowed to grow in the presence of appropriate antibiotic for 24 hours before plating on selective solid medium. Transformants were picked after 4 to 7 days and subcultured in selective SP4 medium. The expression of *M. agalactiae* surface antigen P40 was tested by Western blotting.

### Western blotting and immunodetection of *M. agalactiae* lipoprotein P40

Mycoplasmas grown in SP4 medium were collected by centrifugation at 10,000× g and resuspended in Dulbecco's phosphate-buffered saline (Invitrogen). The protein concentration was determined using the Quick Start Bradford protein assay (Bio-Rad). For Western blotting, total proteins (0.5 µg) were separated by SDS-PAGE using the Mini-Protean II electrophoresis system (Bio-Rad) and transferred to Protran nitrocellulose membranes (Whatman). Membranes were blocked in Tris-buffered saline (TBS) (10 mM TrisHCl, pH 7.4; 150 mM NaCl) containing 5% skim milk for 2 hours, then incubated overnight at 4°C with a sheep anti-P40 serum at a dilution of 1/500 in TBS containing 0.05% Tween 20 and 10% heat-inactivated horse serum (Invitrogen). Western blots were developed using horseradish peroxidase conjugated secondary antibody raised in rabbits (DAKO) and 4-chloro-naphthol as substrate. Sheep serum raised against the *M. agalactiae* surface antigen P80 was used as a control (dilution of 1/200). The anti-P40 and anti-P80 sheep sera were produced by animal immunization with P40 or P80 recombinant proteins, respectively (data not shown).

## Results and Discussion

### High-throughput identification of *M. agalactiae* growth-deficient mutants upon co-cultivation with host cells

Our group has previously reported the construction of a library of *M. agalactiae* knockout mutants together with the development of a high-throughput screening method based on the co-cultivation of *M. agalactiae* mutants with HeLa cells [20]. Since human epithelial cells are not a natural environment for *M. agalactiae*, we subsequently screened the library with two caprine cell lines, which are likely to be more relevant to the normal host-cell interactions involved in infections with this pathogen. The goat mammary epithelial cells, TIGMEC, are likely to be particularly relevant given the predilection of *M. agalactiae* for the mammary gland. HeLa cells were used as a control.

**Figure 1:**
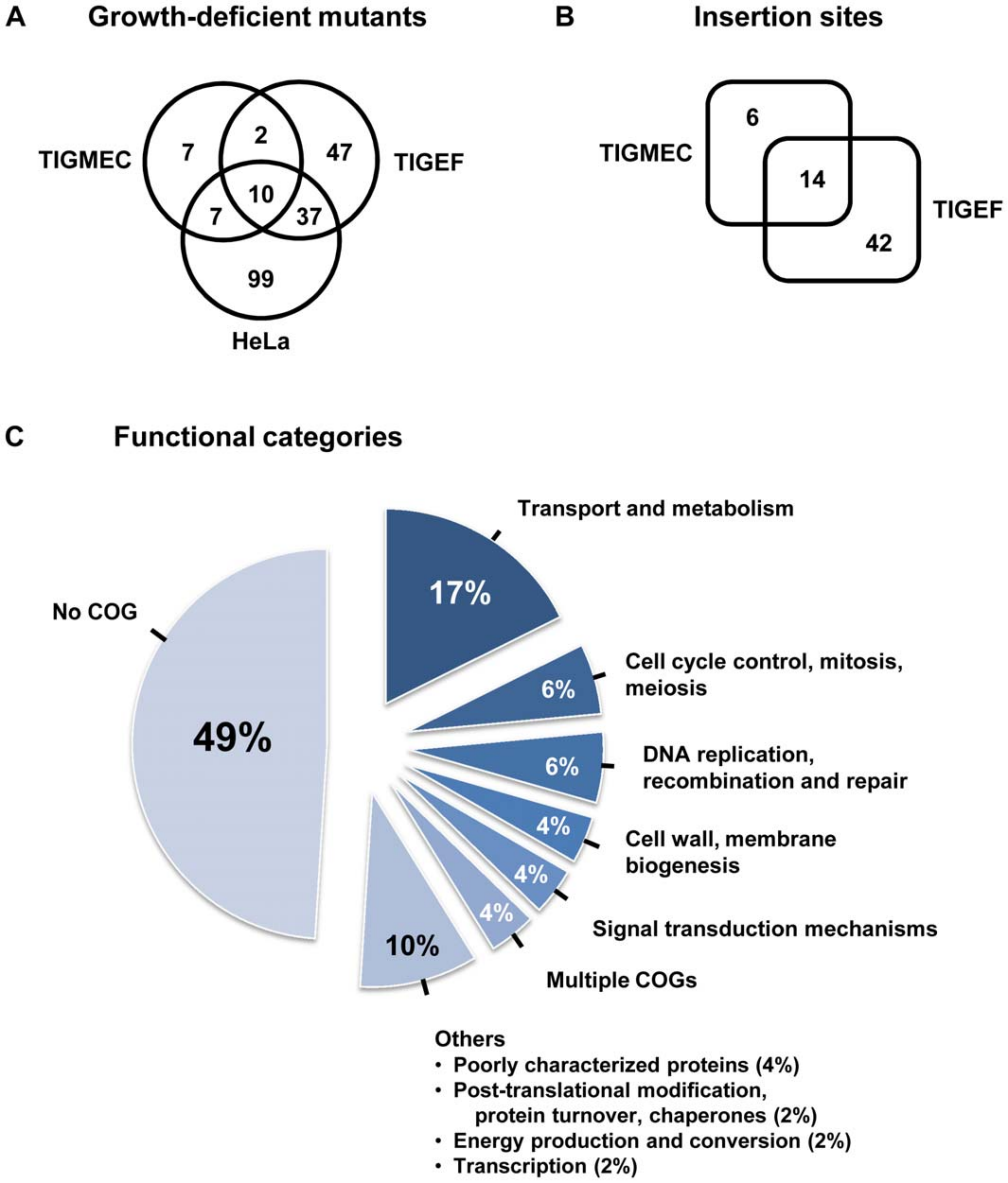
Overall distribution of *M. agalactiae* growth deficient mutants selected after co-cultivation with host cells. (A) Number of mutants selected on goat mammary epithelial cells (TIGMEC), goat embryo fibroblasts (TIGEF) and/or HeLa cells. (B) Number of unique transposon insertion sites identified in mutants selected for their inability to grow on TIGMEC and/or TIGEF. Some mutants had the same insertion site. (C) Distribution in COG categories of the 46 CDSs found disrupted in growth deficient mutants selected on caprine cells (see panel B) [38]. Multiple COG proteins include MAG1490 and MAG2120, which fall into several COG categories (energy production and conversion, C-COG, coenzyme transport and metabolism, H-COG and general function prediction only, R-COG for MAG1490; general function prediction only, R-COG, signal transduction mechanisms, T-COG, transcription, K-COG and replication, recombination and repair, L-COG, for MAG2120).

The ability of individual mutants to grow in the presence of these three cell lines was assessed after 3 days of co-cultivation by directly spotting the cultures onto solid medium and comparing the titers (see Materials and Methods). A total of 209 growth-deficient mutants were selected out of 2,175 tested, using a cut-off value of 10 colonies per plate, which corresponded to a titer of 10^5^ CFU/ml in co-cultures (Table 2). The mutants unable to grow in each cell line are shown in Fig. 1A, with a detailed description of those unable to grow on caprine cells provided in Tables 3 and 4. Some differences were seen between the repertoire of mutants identified as growth deficient on HeLa cells in the current study and those identified in our previous study [20], with only 61% common to both studies. This reflects the limitations of these assays, including some cross contamination between individual mutants stored in 96-well plates. These were found to hamper the selection of mutants with reduced growth capacities in cell culture (data not shown) and to affect the reproducibility of the screening from one experiment to the other.

**Table 3:** *M. agalactiae* CDSs identified by high-throughput screening for their reduced growth capacities on cultured cells.

CDS name^a^	Cells^b^	No. of mutants (no. of insertion sites)^c^	% CDS (orientation)^d^	Gene	Gene product (COG)^e^	Predicted Localization^f^	Detected by MS/MS^g^
MAG0490	F	1 (1)	0.62 (−)		CHP	M	−
MAG0640	F	1 (1)	0.71 (−)	*asnA*	Aspartate-ammonia ligase (E)	C	−
MAG0720	E, F *	2 (2)	0.18 (−)/1.00 (+)	*nifS*	Cysteine desulfurase (E)	C	−
MAG0890	E, F *	2 (1)	0.85 (+)	*hprK*	Hpr kinase phosphorylase (T)	C	+
MAG1180	E, F *	1 (1)	0.95 (−)	*pepP*	XAA-PRO aminopeptidase (E)	C	+
MAG1330	F *	1 (1)	0.31 (−)		CHP DUF285 family, predicted lipoprotein	M	−
MAG1430	E, F *	6 (3)	0.15 (+)/0.84 (+)/0.85 − 0.95 (−)		HP	M	+
MAG1490	F *	1 (1)	0.86 (−)	*ldhD*	D-lactate dehydrogenase (CHR)	IM	+
MAG1500	F	2 (1)	0.56 (+)		Esterase lipase (R)	C	−
MAG1540	F *	1 (1)	0.27 (−)	*tig*	Trigger factor (O)	C	+
MAG1740	F	1 (1)	0.32 (−)	*gidA*	Glucose-inhibited division protein A (D)	C	+
MAG1860	F *	1 (1)	0.63 (−)	*gidB*	Methyltransferase GidB (M)	C	−
MAG1890	F	1 (1)	0.11 (−)		HP	M	+
MAG2110	E	1 (1)	0.08 (+)		Protein phosphatase (T)	C	−
MAG2120	E, F *	1 (1)	0.53 (−)	*pknB*	Serine/threonine-protein kinase (RTKL)	M	+
MAG2540	F *	20 (1)	0.57 (−)		HP, Vpma-like, predicted lipoprotein	M	+
MAG2680	F *	1 (1)	0.60–0.70 (−)		HP	M	+
MAG2870	F *	2 (2)	0.16 (−)/0.72 (+)		CHP, predicted lipoprotein	M	−
MAG2930	E *	1 (1)	0.01 (−)	*atpA*	ATP synthase á chain (C)	IM	+
MAG2960	F *	1 (1)	0.17 (−)		CHP, predicted lipoprotein	M	−
MAG3030	F	3 (1)	0.31 (+)		HP	M	−
MAG3350	F *	1 (1)	0.18 (−)		HP	M	−
MAG3370	F	1 (1)	0.72 (−)		CHP	M	−
MAG3480	F *	1 (1)	0.70 (−)		HP	C	+
MAG3720	E, F	1 (1)	0.10 (+)		CHP	IM	−
MAG3740	F *	1 (1)	0.2–0.4 (+)	*mraZ*	MraZ (S)	IM	−
MAG3790	E, F *	10 (1)	0.95 (−)	*uvrA*	UvrABC system protein A (L)	C	+
MAG3860	F *	1 (1)	ND (−)		CHP	IM	−
MAG4200	F	1 (1)	0.97 (+)		CHP	C	−
MAG4380	F *	3 (2)	0.50–0.75 (−)/0.88 (+)		P115-like ABC transporter ATP binding protein (D)	C	+
MAG4650	E	1 (1)	0.98 (−)		Phosphomannomutase (G)	C	+
MAG4740	F	1 (1)	0.11 (+)		HP, predicted lipoprotein	M	+
MAG4820	E	2 (1)	0.97 (−)		CHP (M)	C	−
MAG4950	E, F *	1 (1)	0.79 (−)		HP, predicted lipoprotein	M	−
MAG5000	F	4 (1)	0.22 (+)		HP	M	−
MAG5150	F *	1 (1)	0.91 (−)		HP, predicted lipoprotein	M	+
MAG5910	F	1 (1)	0.72 (+)		5′ nucleotidase, predicted lipoprotein (F)	M	+
MAG6090	F	1 (1)	0.96 (−)		HP, predicted lipoprotein	M	−
MAG6450	E	1 (1)	0.87 (+)		CHP	C	−
MAG6690	F	1 (1)	0.67 (−)		HP	M	−
MAG6760	F	1 (1)	0.07 (−)	*chrA*	Chromate transport protein (P)	M	−
MAG6770	F *	1 (1)	0.99 (−)	*chrA*	Chromate transport protein (P)	M	+
MAG6870	F	2 (2)	0.60 (−)/0.70 (−)	*dnaX*	DNA polymerase III subunits gamma and tau (L)	C	+
MAG6960	E, F *	1 (1)	0.58 (−)	*apt*	Adenine phosphorybosyltransferase (F)	C	+
MAG7100	F	1 (1)	0.56 (−)	*vpmaZ*	Variable surface lipoprotein D (VpmaZ precursor)	M	+
MAG7200	F	1 (1)	0.74 (+)	*scpB*	Segregation and condensation protein B (K)	C	+

a CDS found disrupted in *M. agalactiae* growth-deficient mutants [6].

b Letters E and F indicate that the *M. agalactiae* growth-deficient mutants were selected on TIGMEC or TIGEF cells, respectively. Asterisks (*) indicate mutants that were also selected during high-throughput screening on HeLa cells.

c For each CDS, the number of mutants identified during the screening on caprine cell lines is indicated, as well as the number of different mini-Tn insertion sites.

d For each CDS, the relative position and the orientation of the inserted transposon are indicated. Mini-Tn insertion sites were determined by direct sequencing of genomic DNA, and their positions were defined based on the published genome sequence (NC_009497).

e Hypothetical proteins (HP) have no homolog outside the species *M. agalactiae*. Conserved hypothetical proteins (CHP) share sequence similarity with proteins of unknown function identified in other *Mollicutes* or other bacteria. COG categories of encoded proteins are indicated in parentheses [38].

f Protein localization was predicted using TMHMM [41], [42]; membrane (M), cytosolic (C), or indirectly linked to the membrane (IM).

g Proteins with peptides detected during proteomic analysis of gene products expressed by *M. agalactiae* strain PG2 in axenic culture are identified by a plus sign (+), while proteins not detected are identified by a minus sign (−) [32].

**Table 4:** *M. agalactiae* NCRs identified by high-throughput screening with host cells.

Name^a^	Cells^b^	No. of mutants (no. of insertion sites)^c^	Size of NCR	Genomic position (orientation)^d^	Genetic environment of NCR^e^
NCR A	F	1 (1)	67 nt	31900 (+)	HP (MAG0310) and HP (MAG0320)
NCR B	E, F *	7 (1)	676 nt	388694 (+)	*ptsG* (MAG3250) and CHP DUF285 family (MAG3260)
NCR C	E, F *	2 (1)	740 nt	402664 (+)	HP (MAG3390) and CHP (MAG3400)
NCR D	F	1 (1)	351 nt	460191 (+)	Pseudogene of CHP (MAG3880) and pseudogene of CHP (MAG3890); vestige of ICEA
NCR E	F *	1 (1)	423 nt	469389 (+)	HP (MAG3950) and CHP (MAG3960); AMIGene CDS prediction of 52 AA (from 469474 to 469319); vestige of ICEA
NCR F	F *	2 (1)	330 nt	473081 (+)	HP (MAG4010) and HP (MAG4020); vestige of ICEA
NCR G	E	1 (1)	802 nt	648734 (−)	*gyrA* (MAG5630) and *hsdS* (MAG 5640); AMIGene CDS prediction of 67 AA (from 648894 to 648694)
NCR H	F	1 (1)	290 nt	761860 (−)	HP (MAG6430) and putative prophage protein ps3 (MAG6440)
NCR I	F *	2 (2)	80 nt	843595 (+)/843634 (+)	*cmk* (MAG7250) and HP (MAG7260)

a NCRs were labeled with a letter based on their position in the genome of *M. agalactiae*.

b Letters E and F indicate NCRs carrying insertions in mutants with reduced growth capacities on TIGMEC and/or TIGEF, respectively. Asterisks (*) indicate mutants that were also selected during high-throughput screening on HeLa cells.

c For each NCR, the number of mutants identified during the screening with caprine cells is indicated, as well as the number of insertion sites.

d Transposon insertion sites were determined by direct sequencing of genomic DNA and their positions were defined based on the published genome sequence (NC_009497). The orientation of the transposon is indicated in parentheses.

e Surrounding CDS and mini-Tn disrupted AMIGene CDS predictions; CDS names are given in parenthesis; vestige of ICEA indicates an NCR located within a 20 kb locus containing a vestige of an integrative conjugative element (ICEA).

Some mutants (27%) displayed reduced growth capacities on multiple cell types, while the majority (73%) exhibited this phenotype on one cell line (Fig. 1A). Interestingly, only a small number of mutants were selected on TIGMEC, compared to the number selected on TIGEF and HeLa cells (Table 2). It is possible that the tolerance of *M. agalactiae* to transposon mutagenesis increases when grown on a cell type similar to its natural environment, but this hypothesis needs to be confirmed with a larger panel of host cell types.

### Mapping of transposon insertion sites in the genome of growth-deficient *M. agalactiae* mutants

Direct genomic sequencing was performed to determine the position of the transposon in mutants found to be inhibited on caprine cell lines. The 110 mutants had 62 unique insertion sites that mapped within 46 different coding sequences (CDS) and 9 non-coding regions (NCR) (Fig. 1B and Tables 3, 4). The examination of DNA sequence chromatograms failed to reveal multiple transposon insertion events in the *M. agalactiae* chromosome (data not shown).

As shown in Figure 1C, most of the disrupted CDSs have been annotated as hypothetical proteins (HP) of unknown function (no COG). Within this category, predicted surface lipoproteins were highly represented 2.1 times more than would be expected [6]. Because of the absence of a cell wall in mycoplasmas, cell surface lipoproteins are thought to be key players in modulating interactions with the host. Interestingly, 20 mutants with a disruption in the same lipoprotein gene (MAG2540) were inhibited on the TIGEF, but not the TIGMEC (Table 3). All had the transposon inserted at the same position, suggesting that they were probably siblings derived from the same parental clone. The repeated selection of this particular mutant suggests a role for MAG2540 in the proliferation of *M. agalactiae* on TIGEF, even though it is dispensable in axenic culture medium and in the presence of TIGMEC. The function of this CDS is unknown, but it is predicted to encode a surface lipoprotein (Vpma-like lipoprotein) with similarities to some domains of the hypervariable Vpma lipoproteins of *M. agalactiae* [24]. Because MAG2540 knockout mutants were also repeatedly selected on HeLa cells, it is possible that, in the absence of specific receptors on target cells such as the TIGMEC, this Vpma-like lipoprotein facilitates binding to ubiquitous structures on mammalian cell surfaces. This implies that growth of *M. agalactiae* in cell cultures requires a close interaction with the cells that is lost in the MAG2540 knockout mutants when growing on TIGEF or HeLa cells. As expected for bacteria with small genomes and reduced metabolic pathways, “transport and metabolism” (COG categories E, F, G, H and P) was the largest functional category involved in interactions between *M. agalactiae* and caprine cells, with 17% of the mutants selected having insertions in genes involved in these functions (Fig. 1C). Since the wild-type is unable to proliferate in cell-culture medium alone [20], it is most likely that these mutants are unable to transport or metabolize some of essential nutrients provided by the cultured cells. The remaining CDSs for which a predicted function had been assigned were distributed across a broad range of functional categories (Fig. 1C), often without any predictable correlation with their role in interactions with caprine cells.

Some of the loci identified in mutants selected on caprine cell lines (Tables 3 and 4), were also identified as being required for growth on HeLa cells, either in this study (Tables 3 and 4) or in our previous study [20]. This suggests that these loci may be involved in general processes mediating interactions between *M. agalactiae* and mammalian cells. The functions encoded by several of these, including DNA repair (*uvrA*), nucleotide metabolism (*apt*), iron-sulfur cluster biosynthesis (*nifS*), and protein folding (*tig*), contribute to stress tolerance and virulence in a number of pathogenic bacteria [25]–[31].

A global proteomic analysis of gene products expressed by *M. agalactiae* strain PG2 in axenic culture [32] detected approximately 50% of the CDSs found to be required for growth on caprine cells (Table 3). Whether this reflects the limited sensitivity of the proteomic approach, or differential expression by *M. agalactiae* under axenic and co-culture conditions is not known.

About 20% of the mutants selected on caprine cells had an insertion in a NCR (Table 4). The importance of NCRs in the biology of *M. agalactiae* remains largely unexplored, but several are likely to include transcriptional promoters and other regulatory regions. Re-examination of the 9 mutated NCRs using the AMIGene annotation tool [33] revealed that two, NCR E and G, had an insertion within short CDSs (Table 4) that had not been annotated previously but that could encode products with a role in host-cell interaction. Interestingly, transcriptional activity in NCRs has been reported for a number of mycoplasmas [34], [35], suggesting an active role for these regions. NCRs D, E and F (Table 4) are part of a 20 kb locus containing a vestige of an integrative conjugative element (ICEA) [6], [36]. The mechanism by which NCRs may regulate the proliferation of *M. agalactiae* in cell culture remains to be elucidated.

Finally, several CDSs required for growth on host cells are conserved in other ruminant mycoplasma species, and/or were involved in the massive horizontal gene transfer (HGT) that occurred between *M. agalactiae*, *M. bovis* and members of the phylogenetically distant mycoides cluster (Table S1). Two CDSs (MAG2870 and MAG6690) were also predicted to have undergone HGT between the avian mycoplasma species *M. synoviae* and *M. gallisepticum* (Table S1). About 70% of these CDSs involved in HGT encode membrane-associated proteins, and thus are likely to play a role in mycoplasma–host interactions. The genome-scale analysis of *M. agalactiae* in cell culture may assist in understanding pathogenic processes involved in other mycoplasma infections.

### The frequent occurrence of promoter regions in the genome of *M. agalactiae* reduces polar effects mediated by integrated transposon sequences

As mentioned above, some CDSs required for growth on cell cultures fell into functional categories (Fig. 1C) that had no obvious correlation with mycoplasma-host interactions. This raised the question of whether growth-deficient phenotypes can result from a polar downstream effect rather than the effect on the gene disrupted by the transposon insertion. The compact mycoplasma genome, which is often organized into highly dense co-linear gene clusters with operon-like structures, suggests that this might be likely [3].

**Figure 2:**
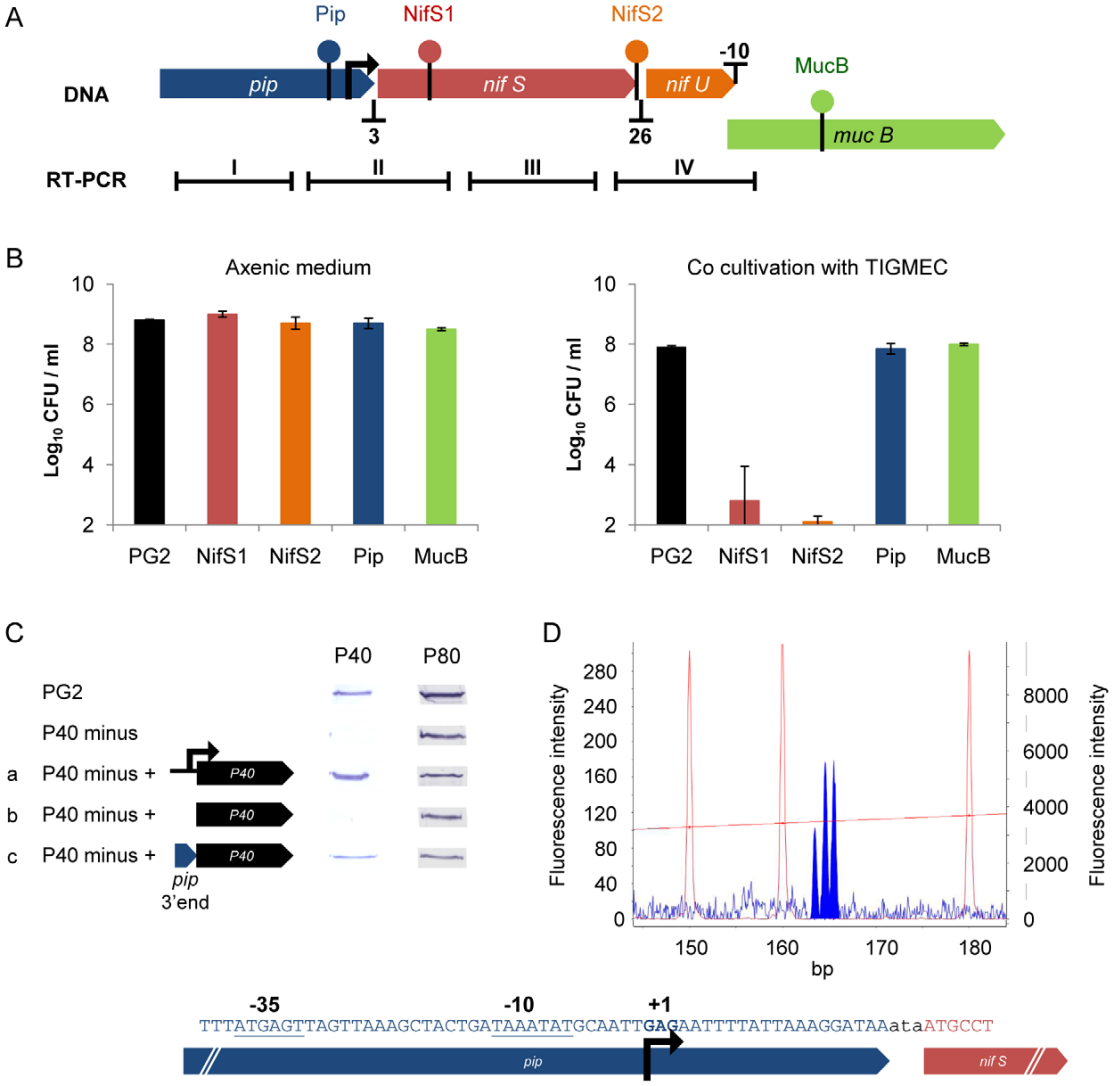
The *nif* locus in *M. agalactiae* is a promoter-less CDS cluster inserted within a transcriptionally active region. (A) Schematic representation of the 4 kbp co-linear gene cluster *pip-nifS-nifU-mucB* indicating the four regions amplified by RT-PCR. Intergenic distances in nucleotides are indicated below. Transposon insertion sites are indicated by filled circles using a different color code for each mutant. RNA regions amplified by RT-PCRs I to IV are indicated by black bars. The promoter identified in the 3′ end of *pip* is indicated by a black arrow. (B) Bar graph indicating the titers obtained, after cultivation in axenic media or in the presence of TIGMEC, of *M. agalactiae* strain PG2 or mutants NifS1, NifS2, Pip and MucB. Titers were determined after 48 h cultivation in axenic medium and after 72 h co-cultivation with TIGMEC. The bars indicate the means of three independent assays, with standard deviations indicated by error bars. (C) Identification of promoter sequences in *M. agalactiae* using a new reporter system. The surface antigen P40-knockout mutant (mutant P40 minus) was complemented using plasmid constructs containing the P40 coding sequence preceded by its own promoter (a), without a promoter (b), or preceded by the 3′ end of *pip* (c). The expression of the P40 antigen was detected by Western blotting. The surface antigen P80 was used as control. (D) Chromatogram obtained after primer extension of total RNA extracted from a *M. agalactiae* PG2 culture with primer 5′-6FAM-NifS1 (Table 1) as described in the Materials and Methods. Red peaks correspond to the GS-400HD ROX internal lane standards and the shaded blue peaks to the primer extension product. The size is indicated on the X-axis in base pairs. The peak height indicates the fluorescence intensity (arbitrary units) with GS-400HD ROX fluorescence measured on the right Y-axis and FAM fluorescence on the left Y-axis. The position of the transcriptional start point in CDS MAG0710 (*pip*) identified by primer extension experiments is indicated. Putative −10 and −35 regions are underlined. The transcriptional start points are indicated in bold.

To evaluate the potential influence of the mini-Tn insertions on gene expression from these operon-like structures, we analyzed the 4 kb co-linear gene cluster *pip-nifS-nifU-mucB* (MAG0710 to MAG0740) (Fig. 2). This cluster was of particular interest because: (i) *nifS* has been shown to be essential for proliferation of *M. agalactiae* on cell cultures, and two knockout mutants, NifS1 and NifS2, were identified by high-throughput screening on caprine and human cells (Table 3); and (ii) these co-linear genes are likely to be co-transcribed by a promoter located upstream of *pip*, based on genome data indicating short intergenic distances (Fig. 2A). Indeed, the operon-like structure of the *pip-nifS-nifU-mucB* cluster was further supported by our transcriptional analyses, which detected overlapping transcripts by RT-PCR (Fig. 2A). Two mutants, Pip and MucB, with a transposon inserted into the corresponding genes, were searched for and found in the mutant library (see Materials and Methods), but both are able to grow on all three cell lines. When tested individually with TIGMEC, both mutants had the wild-type phenotype (Fig. 2B). This result was surprising, at least for the Pip mutant, which was expected to have a similar phenotype to that of the NifSs mutants because of the predicted polar effect of the transposon insertion (Fig. 2A).

RT-PCR amplification using oligonucleotide primers bracketing the mini-Tn integration site in mutants Pip, NifS1, and NifS2 did not detect mRNA (RT-PCR II in mutants Pip and NifS1, and RT-PCR IV in mutant NifS2), but, unexpectedly, transcripts were detected downstream of the mini-Tn (RT-PCR III and IV in mutants Pip and NifS1), suggesting that there may be a promoter within *pip* or the mini-Tn. The presence of a promoter within the integrated mini-Tn sequences was suggested by the identification of RNA transcripts that overlapped the 3′ end of the mini-Tn and *nifS* in Pip and NifS1 mutants (data not shown). The extension of transcription beyond the 3′ end of the mini-Tn was probably responsible for the expression of downstream genes in knockout mutants of *M. agalactiae* and may have influenced the orientation of the mini-Tn in the mutant library [20]. Although promoter sequences in the mini-Tn remain to be formally demonstrated, constitutive gene expression from the 3′ end of the mini-Tn can also be viewed as another outcome of transposon mutagenesis. Experiments are in progress to determine the role of this mechanism in mutants unable to grow in cell cultures that harbor a transposon within a NCR.

Recent studies on *Mycoplasma genitalium* have revealed that the genome of this human pathogen is actively transcribed, and contains multiple cryptic promoters [34]. This led us to further explore transcriptional activity within the *pip-nifS-nifU-mucB* cluster, and to examine the promoter activity at the 3′ end of the *pip* gene. A reporter system was developed to detect promoter sequences in *M. agalactiae* (Fig. 2C). Lipoprotein P40 (MAG2410) is a surface antigen of *M. agalactiae*, the expression of which can be detected using specific antibodies. The successful complementation of a *M. agalactiae* mutant, P40 minus, harboring a transposon within the P40 lipoprotein gene, with a plasmid expressing P40, led us to develop a reporter system to detect promoter activity. Western blotting analyses confirmed that the 157 nucleotides at the 3′ end of the *pip* gene, when inserted at the 5′ end of the P40 lipoprotein gene, promote complementation of the P40 minus mutant.

Finally, the transcriptional start point at the 3′ end of the *pip* gene was mapped by primer extension using the oligonucleotide 5′-6FAM-NifS1 (Table 1). A major extension product with a size of between 163 and 165 nucleotides was obtained, indicating that the transcriptional start point was 31 nucleotides upstream of the stop codon of the *pip* gene (Fig. 2D). The detection of multiple extension products differing in length by a single nucleotide is a common finding in mycoplasma promoters [34], [37].

The genomes of mycoplasmas are characterized by a low G+C content. This particular feature may favor the occurrence of primary or secondary promoter regions in the genome of *M. agalactiae*, even within coding sequences such as this promoter within the 3′ end of the *pip* gene. The high density of promoters within the genome of *M. agalactiae* may have important evolutionary consequences, facilitating chromosomal rearrangements and gene shuffling in these rapidly evolving organisms.

### Gene involved in the interaction of *M. agalactiae* with host cells may have been hitchhiking across evolution

**Figure 3:**
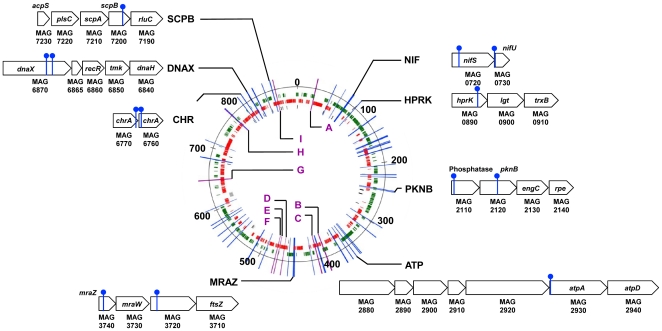
Genomic loci carrying transposon insertions in *M. agalactiae* mutants displaying reduced growth capacities in cell culture. Map of the 62 genomic regions found to be disrupted in *M. agalactiae* mutants selected on TIGMEC and/or TIGEF cells, produced using the Artemis genome browser and annotation tool [39]. Insertion sites found within CDS regions are indicated by a blue bar, while intergenic regions are designated by a letter code and a purple bar. Short CDS clusters are designated by capital letters and a schematic illustration of the locus, in which insertion sites are indicated by a filled circle on top of a blue bar. Genes are labeled. CDSs for hypothetical proteins of unknown function are labeled with their gene number. Genomic distances are indicated in kbp. *M. agalactiae* CDSs are colored in green (positive) or red (negative) on the chromosome to indicate their orientation. Non-coding RNAs are colored in black.

Although polar effects resulting from the disruption of co-transcribed gene clusters cannot be ruled out, the identification of multiple transposon insertion sites in the same region suggests that important factors mediating the interaction between *M. agalactiae* and host cells may map to these loci. Among the 62 loci identified after screening with host cells, six loci mutants had insertions in the same CDS or NCR, but at different positions (Tables 3 and 4). CDSs with multiple insertion sites included two HP predicted to be exposed on the cell surface (MAG1430 and MAG2870), and cytosolic proteins with homologies with cysteine desulfurase (MAG0720), the P115-like ABC transporter ATP binding protein (MAG4380), and DNA polymerase III subunits gamma and tau (MAG6870). Only one NCR (NCR I) was found containing two different insertions that inhibited the capacity of *M. agalactiae* to grow on host cells. Interestingly, CDSs MAG0720 (*nifS*) and MAG6870 (*dnaX*) each belong to co-linear gene clusters (Fig. 3) that are highly conserved in *Mollicutes* (Fig. S1, S2, and S3). The remarkable conservation of these two gene clusters led us to examine the co-localization of the 46 CDS identified as being required for growth of *M. agalactiae* on cultured cells, and to compare the synteny of these CDSs using complete genome sequences of mycoplasmas available in public databases. A total of 8 highly conserved co-linear gene clusters were identified (Fig. 3 and Fig. S1, S2, and S3). Several of these clusters encode proteins predicted to be involved in specific functions, including [Fe-S] cluster biosynthesis (NIF), chromate transport (CHR), cell division (MRAZ) and DNA replication, recombination and repair (DNAX). The potential role of these CDS clusters in the interaction between *M. agalactiae* and host cells was further supported by the similar effect of transposon insertions at different positions in these CDS clusters (NIF, PKNB, MRAZ, CHR and DNAX in Fig. 3). The essential role of the NIF locus in the interaction between *M. agalactiae* and host cells has been demonstrated previously by complementation studies [20].

The location of these CDS clusters can differ considerably from one mycoplasma species to another, even within the same phylogenetic group (Fig. S1, S2, and S3). In *M. agalactiae*, promoter sequences initiating the transcription of *nifS* were located outside of the NIF locus, with a sequence identified within the 3′ end of the *pip* gene (see above). Interestingly, the *pip* gene does not always co-localize with the NIF locus in other mycoplasma species, suggesting that this locus is inserted within transcriptionally active regions. Thus, it is tempting to speculate that some of the genes involved in the interaction between *M. agalactiae* and host cells may have been hitchhiking across evolution for transcriptionally active regions of the mycoplasma chromosome. Further evidence is needed to support this hypothesis.

In conclusion, cell culture provides a simple and efficient screening system for genome-scale analysis of mycoplasma loci contributing to host-pathogen interactions. This global approach, when combined with *in vivo* studies, can be an efficient strategy for identifying key factors involved in mycoplasma virulence and host-colonization, as well as a way to understand pathogenic processes involved in disease caused by these unconventional pathogens. The absence of small animal models for *M. agalactiae* and other ruminant mycoplasmal pathogens has been a significant bottleneck for functional genomics studies using large mutant libraries. High-throughput screening of more than 2000 individual clones by co-culture with host cells revealed 62 loci in the genome of *M. agalactiae* that were required for growth in this environment. The relevance of these loci in the biology of *M. agalactiae* when it replicates in its natural host remains to be determined, but experimental infections in lactating ewes have confirmed the essential role played by *nifS* in host-colonization (data not shown). Since the specific cell line was shown to influence which mutants were selected, comparative studies with different cell types, including differentiated monolayer cultures and cellular players involved in the immune response to infection, may provide further information about the interactions of mycoplasmas in these different cellular environments. Finally, these results provide an experimental framework for the development of control strategies, based on attenuated live vaccines, against mycoplasmosis in ruminant species.

## Supporting Information

Figure S1 **Genomic position of short CDS clusters NIF, HPRK, MRAZ and CHR in mycoplasma species with sequenced genomes.** Gene organization of the conserved CDS clusters identified during the screening was analyzed using the Microbial Genome Database for Comparative Analysis software [40]. Homologues genes are indicated by the same color code and a black bar. Three examples were chosen for each phylogenetic group. In the case of the CHR locus, no homolog was identified in the Pneumoniae group. MA: *M. agalactiae*, MMOB: *M. mobile*, MYPU: *M. pulmonis*, MLEA: *M. leachii*, MmmSC: *Mycoplasma mycoides* subsp. *mycoides* SC, MCAP: *M. capricolum* subsp. *capricolum*, UPAR: *U. parvum*, UURE: *U. urealyticum*, MGAL: *M. gallisepticum*.
(TIF)

Figure S2 **Genomic position of short CDS clusters PKNB and ATP in mycoplasma species with sequenced genomes.** Gene organization of the conserved CDS clusters identified during the screening was analyzed using the Microbial Genome Database for Comparative Analysis software [40]. Homologues genes are indicated by the same color code and a black bar. Three examples were chosen for each phylogenetic group. MA: *M. agalactiae*, MMOB: *M. mobile*, MYPU: *M. pulmonis*, MLEA: *M. leachii*, MmmSC: *Mycoplasma mycoides* subsp. *mycoides* SC, MCAP: *M. capricolum* subsp. *capricolum*, UPAR: *U. parvum*, UURE: *U. urealyticum*, MGAL: *M. gallisepticum*.
(TIF)

Figure S3 **Genomic position of short CDS clusters DNAX and SCPB in mycoplasma species with sequenced genomes.** Gene organization of the conserved CDS clusters identified during the screening was analyzed using the Microbial Genome Database for Comparative Analysis software [40]. Homologues genes are indicated by the same color code and a black bar. Three examples were chosen for each phylogenetic group. MA: *M. agalactiae*, MMOB: *M. mobile*, MYPU: *M. pulmonis*, MLEA: *M. leachii*, MmmSC: *Mycoplasma mycoides* subsp. *mycoides* SC, MCAP: *M. capricolum* subsp. *capricolum*, UPAR: *U. parvum*, UURE: *U. urealyticum*, MGAL: *M. gallisepticum*.
(TIF)

Table S1 **Degree of homology of CDS disrupted in** ***M. agalactiae*** **mutants with other ruminant mycoplasma species.** ^a^CDS found disrupted in *M. agalactiae* growth-deficient mutants [6]. ^b^Hypothetical proteins (HP) have no homolog outside the *M. agalactiae* species. Conserved hypothetical proteins (CHP) share sequence similarity with proteins of unknown function identified in mollicutes or other bacteria. COG categories of encoded proteins are indicated in parenthesis [38]. ^c^Protein localization was predicted using TMHMM [40]; membrane (M), cytosolic (C), or indirectly linked to the membrane (IM). ^d^Genes supposed to have undergone horizontal gene transfer (HGT) between M. agalactiae and mycoplasmas from the mycoides cluster [6] are indicated by a plus sign (+). Genes displaying homologies with sequences in *M. gallisepticum* or *M. synoviae* genomes and supposed to have undergone HGT between these two species are identified by a cross sign (x) [6]. ^e^Percentages of identity and similarity were determine by local BLAST using Molligen [42]. ^f^MCAP stands for *M. capricolum subsp. capricolum*.
(DOC)
