# Supplementary figures and images for: Genome-Scale Analysis of Mycoplasma agalactiae Loci Involved in Interaction with Host Cells
Source: PLoS One. 2011 Sep 23;6(9):e25291. doi: 10.1371/journal.pone.0025291 (PMC3179502; doi:10.1371/journal.pone.0025291)

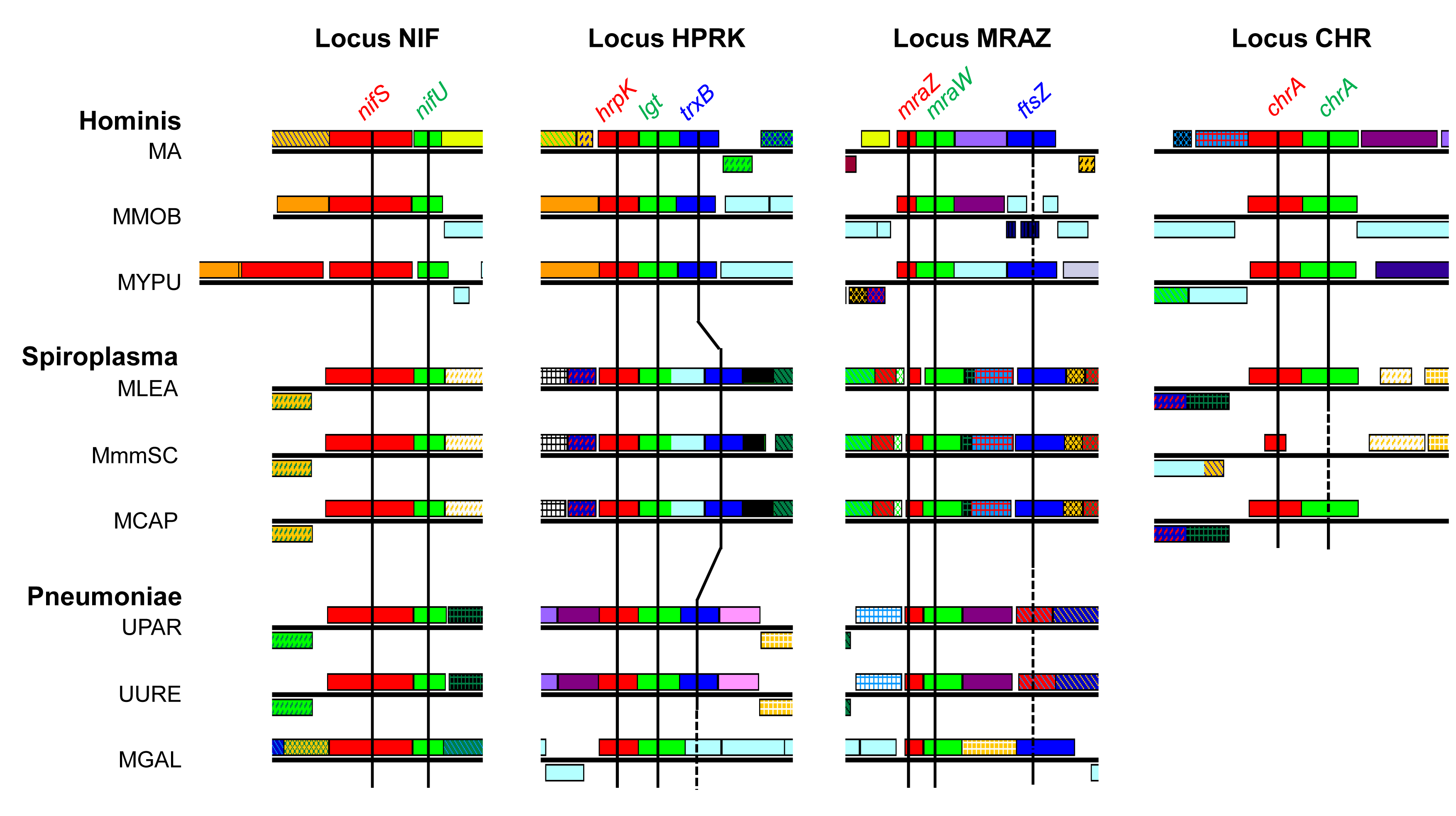

Supplement: Figure S1 — Genomic position of short CDS clusters NIF, HPRK, MRAZ and CHR in mycoplasma species with sequenced genomes. Gene organization of the conserved CDS clusters identified during the screening was analyzed using the Microbial Genome Database for Comparative Analysis software [40]. Homologues genes are indicated by the same color code and a black bar. Three examples were chosen for each phylogenetic group. In the case of the CHR locus, no homolog was identified in the Pneumoniae group. MA: M. agalactiae, MMOB: M. mobile, MYPU: M. pulmonis, MLEA: M. leachii, MmmSC: Mycoplasma mycoides subsp. mycoides SC, MCAP: M. capricolum subsp. capricolum, UPAR: U. parvum, UURE: U. urealyticum, MGAL: M. gallisepticum. (TIF) [file pone.0025291.s001.tif]

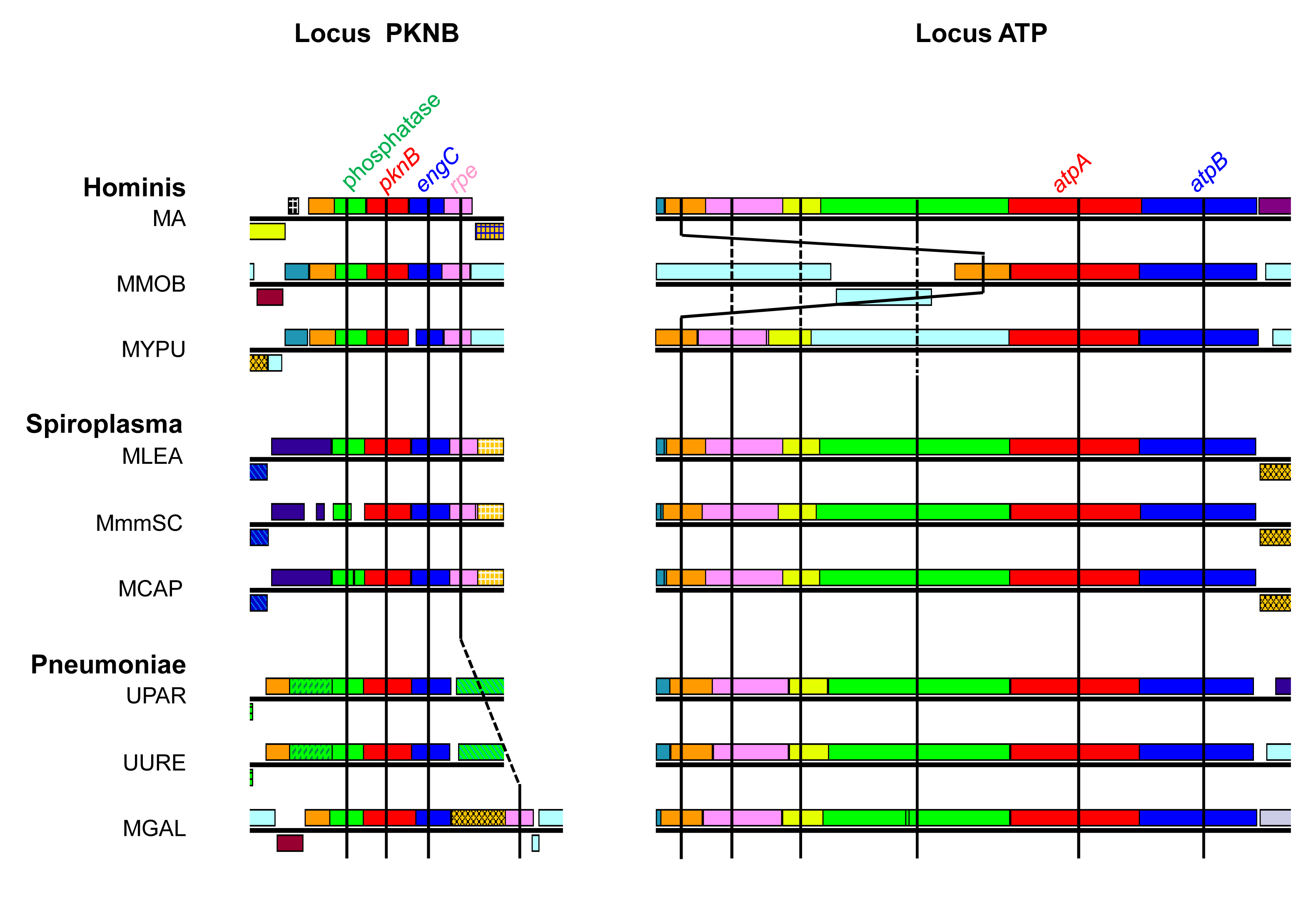

Supplement: Figure S2 — Genomic position of short CDS clusters PKNB and ATP in mycoplasma species with sequenced genomes. Gene organization of the conserved CDS clusters identified during the screening was analyzed using the Microbial Genome Database for Comparative Analysis software [40]. Homologues genes are indicated by the same color code and a black bar. Three examples were chosen for each phylogenetic group. MA: M. agalactiae, MMOB: M. mobile, MYPU: M. pulmonis, MLEA: M. leachii, MmmSC: Mycoplasma mycoides subsp. mycoides SC, MCAP: M. capricolum subsp. capricolum, UPAR: U. parvum, UURE: U. urealyticum, MGAL: M. gallisepticum. (TIF) [file pone.0025291.s002.tif]

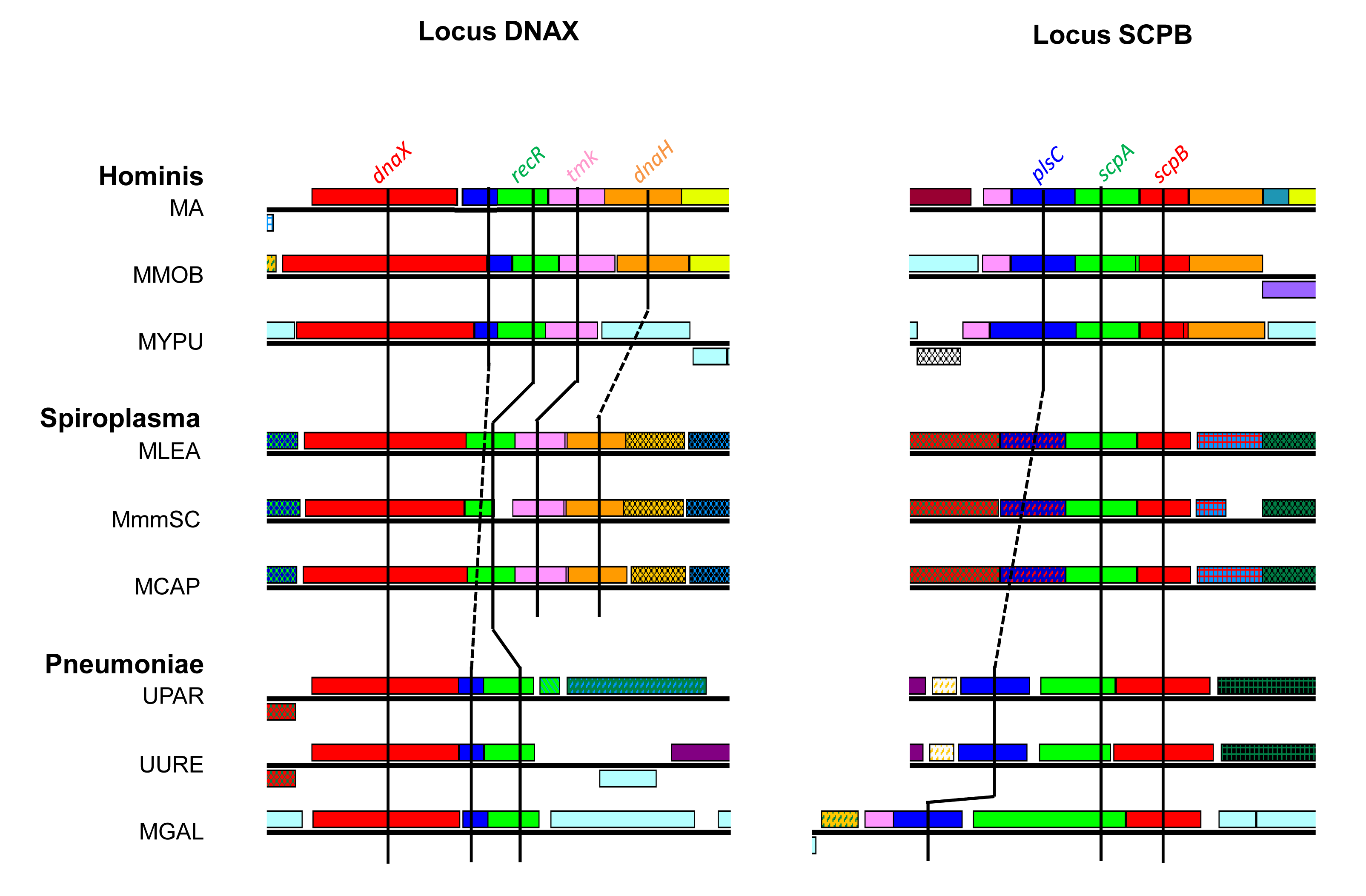

Supplement: Figure S3 — Genomic position of short CDS clusters DNAX and SCPB in mycoplasma species with sequenced genomes. Gene organization of the conserved CDS clusters identified during the screening was analyzed using the Microbial Genome Database for Comparative Analysis software [40]. Homologues genes are indicated by the same color code and a black bar. Three examples were chosen for each phylogenetic group. MA: M. agalactiae, MMOB: M. mobile, MYPU: M. pulmonis, MLEA: M. leachii, MmmSC: Mycoplasma mycoides subsp. mycoides SC, MCAP: M. capricolum subsp. capricolum, UPAR: U. parvum, UURE: U. urealyticum, MGAL: M. gallisepticum. (TIF) [file pone.0025291.s003.tif]
