# Supplementary material for: Genome-Scale Analysis of Mycoplasma agalactiae Loci Involved in Interaction with Host Cells
Source: PLoS One. 2011 Sep 23;6(9):e25291. doi: 10.1371/journal.pone.0025291 (PMC3179502; doi:10.1371/journal.pone.0025291)
Supplement: Table S1 — Degree of homology of CDS disrupted in M. agalactiae mutants with other ruminant mycoplasma species. aCDS found disrupted in M. agalactiae growth-deficient mutants [6]. bHypothetical proteins (HP) have no homolog outside the M. agalactiae species. Conserved hypothetical proteins (CHP) share sequence similarity with proteins of unknown function identified in mollicutes or other bacteria. COG categories of encoded proteins are indicated in parenthesis [38]. cProtein localization was predicted using TMHMM [40]; membrane (M), cytosolic (C), or indirectly linked to the membrane (IM). dGenes supposed to have undergone horizontal gene transfer (HGT) between M. agalactiae and mycoplasmas from the mycoides cluster [6] are indicated by a plus sign (+). Genes displaying homologies with sequences in M. gallisepticum or M. synoviae genomes and supposed to have undergone HGT between these two species are identified by a cross sign (x) [6]. ePercentages of identity and similarity were determine by local BLAST using Molligen [42]. fMCAP stands for M. capricolum subsp. capricolum. (DOC) [file pone.0025291.s004.doc]

| **CDS name ^a^** | **Gene** | **Gene product** | **Predicted** | **HGT ^d^** | **Homolog in** | **% identity /** | **Homolog in** | **% identity /** |
| --- | --- | --- | --- | --- | --- | --- | --- | --- |
|  |  | **(COG) ^b^** | **localization ^c^** |  | ***M. bovis*** | **% similarity ^e^** | **MCAP ^f^** | **% similarity ^e^** |
| MAG0490 |  | CHP | M |  | MBOVPG45_0056 | 64/85 | - | 0 |
| MAG0640 | *asnA* | Aspartate-ammonia ligase (E) | C | + | MBOVPG45_0071 | 88/94 | MCAP0817 | 71/83 |
| MAG0720 | *nifS* | Cysteine desulfurase (E) | C |  | MBOVPG45_0081 | 79/89 | MCAP0469 | 33/54 |
| MAG0890 | *hprK* | Hpr kinase phosphorylase (T) | C |  | MBOVPG45_0100 | 90/96 | MCAP0777 | 33/52 |
| MAG1180 | *pepP* | XAA-PRO aminopeptidase (E) | C |  | MBOVPG45_0127 | 93/96 | MCAP0341 | 38/61 |
| MAG1330 |  | CHP DUF285 family, predicted lipoprotein | M | + | MBOVPG45_0425 | 34/49 | MCAP0300 | 40/50 |
| MAG1430 |  | HP | M |  | MBOVPG45_0157 | 73/84 | - | 0 |
| MAG1490 | *ldhD* | D lactate dehydrogenase (CHR) | IM | + | MBOVPG45_0163 | 94/95 | - | 0 |
| MAG1500 |  | Esterase lipase (R) | C | + | MBOVPG45_0164 | 83/89 | MCAP0606 | 51/68 |
| MAG1540 | *tig* | Trigger factor (O) | C |  | MBOVPG45_0171 | 81/87 | MCAP0517 | 29/50 |
| MAG1740 | *gidA* | Glucose inhibited division protein A (D) | C |  | MBOVPG45_0675 | 93/96 | MCAP0856 | 48/66 |
| MAG1860 | *gidB* | Methyltransferase GidB (M) | C |  | MBOVPG45_0657 | 80/88 | MCAP0807 | 34/50 |
| MAG1890 |  | HP | M |  | - | 0 | - | 0 |
| MAG2110 |  | Protein phosphatase (T) | C |  | MBOVPG45_0630 | 85/91 | MCAP0264 | 36/50 |
| MAG2120 | *pknB* | Serine/threonine-protein kinase (RTKL) | M |  | MBOVPG45_0629 | 81/88 | MCAP0262 | 29/47 |
| MAG2540 |  | HP, Vpma like, predicted lipoprotein | M |  | - | 0 | - | 0 |
| MAG2680 |  | HP | M |  | MBOVPG45_0550 | 64/71 | - | 0 |
| MAG2870 |  | CHP, predicted lipoprotein | M | + x | MBOVPG45_0376 | 74/84 | MCAP0350 | 40/59 |
| MAG2930 | *atpA* | ATP synthase α chain (C) | IM | + | MBOVPG45_0382 | 95/98 | MCAP0358 | 74/87 |
| MAG2960 |  | CHP, predicted lipoprotein | M | + | MBOVPG45_0385 | 66/79 | MCAP0072 | 45/63 |
| MAG3030 |  | HP | M |  | - | 0 | - | 0 |
| MAG3350 |  | HP | M |  | - | 0 | - | 0 |
| MAG3370 |  | CHP | M | + | MBOVPG45_0480 | 42/62 | MCAP0568 | 36/56 |
| MAG3480 |  | HP | C |  | MBOVPG45_0439 | 56/71 | - | 0 |
| MAG3720 |  | CHP | IM |  | MBOVPG45_0461 | 70/83 | - | 0 |
| MAG3740 | *mraZ* | MraZ (S) | IM |  | MBOVPG45_0463 | 96/98 | MCAP0387 | 28/57 |
| MAG3790 | *uvrA* | UvrABC system protein A (L) | C |  | MBOVPG45_0470 | 95/97 | MCAP0774 | 56/73 |
| MAG3860 |  | CHP | IM | + | MBOVPG45_0479 | 96/97 | MCAP0571 | 52/70 |
| MAG4200 |  | CHP | C |  | - | 0 | - | 0 |
| MAG4380 |  | P115 like ABC transporter ATP binding protein (D) | C |  | MBOVPG45_0520 | 87/92 | MCAP0495 | 44/65 |
| MAG4650 |  | Phosphomannomutase (G) | C |  | MBOVPG45_0350 | 82/92 | MCAP0756 | 37/56 |
| MAG4740 |  | HP, predicted lipoprotein | M |  | MBOVPG45_0341 | 45/62 | - | 0 |
| MAG4820 |  | CHP (M) | C |  | MBOVPG45_0333 | 79/90 | MCAP0866 | 25/46 |
| MAG4950 |  | HP, predicted lipoprotein | M | + | MBOVPG45_0320 | 73/85 | MCAP0723 | 38/57 |
| MAG5000 |  | HP | M |  | MBOVPG45_0316 | 56/69 | - | 0 |
| MAG5150 |  | HP, predicted lipoprotein | M |  | MBOVPG45_0298 | 63/77 | - | 0 |
| MAG5910 |  | 5' nucleotidase, predicted lipoprotein (F) | M |  | MBOVPG45_0690 | 88/94 | - | 0 |
| MAG6090 |  | HP, predicted lipoprotein | M |  | MBOVPG45_0710 | 42/63 | - | 0 |
| MAG6450 |  | CHP | C | + | MBOVPG45_0500 | 60/77 | MCAP0494 | 61/73 |
| MAG6690 |  | HP | M | x | MBOVPG45_0767 | 82/88 | - | 0 |
| MAG6760 | *chrA* | Chromate transport protein (P) | M |  | MBOVPG45_0773 | 69/73 | MCAP0198 | 33/46 |
| MAG6770 | *chrA* | Chromate transport protein (P) | M |  | MBOVPG45_0774 | 67/73 | MCAP0197 | 41/60 |
| MAG6870 | *dnaX* | DNA polymerase III subunits gamma and tau (L) | C |  | MBOVPG45_0785 | 81/89 | MCAP0008 | 42/60 |
| MAG6960 | *apt* | Adenine phosphorybosyltransferase (F) | C |  | MBOVPG45_0796 | 94/98 | MCAP0497 | 46/66 |
| MAG7100 | *vpmaZ* | Variable surface lipoprotein D (VpmaZ precursor) | M |  | - | 0 | - | 0 |
| MAG7200 | *scpB* | Segregation and condensation protein B (K) | C |  | MBOVPG45_0832 | 96/98 | MCAP0601 | 26/52 |
